# High Concordance between Self-Reported Adherence, Treatment Outcome and Satisfaction with Care Using a Nine-Item Health Questionnaire in InfCareHIV

**DOI:** 10.1371/journal.pone.0156916

**Published:** 2016-06-16

**Authors:** Gaetano Marrone, Åsa Mellgren, Lars E. Eriksson, Veronica Svedhem

**Affiliations:** 1 Department of Infectious Diseases and Clinical Virology, Karolinska University Hospital, Stockholm, Sweden; 2 Department of Public Health Sciences, Karolinska Institutet, Solna, Sweden; 3 Clinic of Infectious Diseases, South Älvsborg Hospital, Borås, Sweden; 4 Department of Learning, Informatics, Management and Ethics, Karolinska Institutet, Solna, Sweden; 5 Department of Infectious Diseases, Karolinska University Hospital, Stockholm, Sweden; 6 School of Health Sciences, City University London, London, United Kingdom; 7 Unit of Infectious Diseases, Department of Medicine Huddinge, Karolinska Institutet, Stockholm, Sweden; Azienda ospedaliero-universitaria di Perugia, ITALY

## Abstract

**Background:**

In this cross-sectional study we present an integrated analysis of a self-reported Health Questionnaire and socio-demographic and treatment outcome data from the national Swedish HIV cohort, InfCareHIV.

**Objectives:**

To evaluate the Health Questionnaire and identify the main determinants of adherence.

**Methods:**

A total of 2,846 patients answered a nine-item disease-specific Health Questionnaire between 2012 and 2014, corresponding to 44% of all active patients in the national InfCareHIV cohort. The questionnaire assessed patient related outcome measures (PROMs) regarding health and antiretroviral treatment (ART) and patient related experience measures (PREMs) regarding involvement in care and satisfaction with the care provider.

**Result:**

We found the Health Questionnaire to be valid and reliable when used in ordinary clinical practice. There was a high concordance between self-reported adherence to ART in the past seven days and treatment outcome, with 94% of patients who reported optimal adherence having a viral load <50 copies/ml. The main determinants of optimal adherence were heterosexual transmission path, being born in Sweden, being male, not reporting experience of ART side effects and being fully satisfied with care.

**Conclusion:**

The nine-item Health Questionnaire can identify patients at risk of treatment failure, those in need of clinical assessment of adverse events and those with impaired physical health.

## Background

New evidence shows that individuals with HIV who are diagnosed early in the course of infection and who have access and good adherence to antiretroviral treatment (ART) have an estimated median survival of 35 to 45 years [1,2]. Since life expectancy is increasing, it is of great importance to monitor the main determinants of adherence to ART, patients’ satisfaction with care and how HIV impacts on quality of life (QoL). In some parts of the world [3] including Sweden [4], QoL in HIV-infected individuals is reported to be significantly lower than in the general population. Factors negatively influencing health-related QoL are depressive symptoms, incapacity to work, dissatisfaction with the patient-physician relationship, neurocognitive dysfunctioning, non-adherence [3,5] and HIV-related stigma [6]. One way to assess a patient’s health-related QoL is through patient reported outcome measures (PROMs) collected using self-completed questionnaires. PROMs are used to systematically collect information about self-reported health in a group of patients, to evaluate the outcome of care related to patient reported experienced measures (PREMs), to compare treatment effects on subjective health, and as a tool for identifying the risk groups towards which interventions should be directed. When analyzing PROMs and PREMs at individual or group level it is important to be aware of the possible difference between a medical assessment and the patient’s view.

### Adherence

Adherence to ART is one of the main determinants of treatment success and non-adherence is associated with lower quality of life [5,7], progression to AIDS [8] and mortality [9,10]. Self-reported adherence with a mean recall period of seven days is the most frequently used measure of adherence [11] and in most studies self-reported adherence is significantly related to virological [11,12] and immunological outcomes [13]. Depressive symptoms, current drug abuse, concerns about ART, self-reported adverse drug reactions [14,15] and impaired neurocognitive functioning [16,17] are among some of the factors that may negatively impact on adherence whereas high adherence self-efficacy, trust and satisfaction with the care provider and lower daily dosing correlate with improved adherence to ART [9,18].

This cross-sectional study, called the Health Questionnaire study, combines the analysis of treatment outcome data from the national Swedish HIV cohort, InfCareHIV, using a self-reported nine-item Health Questionnaire administered to HIV patients in Sweden before meeting the HIV-Team between 2012 and 2014. The results are shown in real time in the decision tool of InfCareHIV. The purpose was to evaluate the Health Questionnaire and study the association between socio-demographic data, self-reported adherence, physical health, psychological wellbeing, satisfaction and involvement with care, and treatment outcome defined by plasma HIV-RNA level. The aim was also to identify the main determinants of adherence and possibilities for interventional improvement in order to address unmet needs for individual patients and identified groups of patients with HIV. In addition, a longitudinal substudy was performed among patients who were asked to complete the questionnaire more than once during the study period in order to explore the determinants of changes in adherence patterns over time.

## Material and Methods

### InfCare HIV

In Sweden, >99% of individuals with known HIV infection are followed longitudinally from the date of their diagnosis through InfCareHIV. The InfCareHIV is a Swedish National Quality Assurance Registry, including a database and a clinical support tool, which has been implemented in all 30 HIV care centers in Sweden since 2008. InfCareHIV contains socio-demographic (gender, age, country of origin, estimated country of transmission, route of transmission) and biological data (date of first positive HIV serology, CD4 T-cell count, plasma and CSF HIV-RNA and HCV and HBV serostatus), ART history and information about HIV drug resistance. Patient data is transferred in real time to the clinical support tool accessed by the HIV team. InfCareHIV contains data on 10,064 patients from 1995 to 2015. Data from before 2008 have been partially added retrospectively. In this study, the following variables from InfCareHIV were used: gender, route of transmission, age, country of birth, country of transmission, ART and Viral Load (VL). ART was dichotomized as treatment ongoing or not ongoing at time of response to the questionnaire. Treatment outcome was assessed for patients treated for more than 6 months and was defined as success for patients with VL < 50 copies/ml or failure for VL >50 copies/ml. VL from sampling performed closest to the date of completion of the Health Questionnaire was selected; the mean time was 46 days.

### Health Questionnaire

Between 2008 and 2011, a project team developed a Swedish and English version of a disease-specific self-reported Health Questionnaire to assess PROMs and PREMs in the InfCareHIV cohort. Special efforts were taken to put together a project team with members representing a wide range of disciplines, all active in HIV care and treatment, to ensure content validity of the questionnaire. To ensure face validity, acceptability and feasibility, preliminary versions were used in ordinary clinical practice; experiences were regularly discussed and successive improvements were made during the period 2008 to 2011.

Since 2012, this self-reported nine-item Health Questionnaire was integrated into InfCareHIV as an annual health survey. The final version of the Health Questionnaire consists of nine items (of which the participants answer a minimum of six and a maximum of nine items depending on their treatment and experience of adverse events status) is presented in Supporting Information(S1 Fig). Items 1–3 cover satisfaction with physical, psychological and sexual health. Questions were adapted from the Life-Satisfaction scale (Li-Sat) [19]. For patients on ART (Item 4a), the severity of drug induced adverse events (Item 4c) were assessed using responses on a five-point Likert scale by patients who had reported experiencing such side effects (Item 4b). Information about self-reported ART adherence was collected by asking patients to state how many doses she or he had missed in the past week (0, 1–2, 3 or more doses) (Item 4d). Feelings of involvement (Item 5) and satisfaction with care (Item 6) were also assessed using Likert scales. Item 5 was developed from a Swedish self-assessment used in the systematic evaluation of psychiatric care in western Sweden and adapted to Swedish HIV care [20]. Item 6 was based on the results of a literature review of PREMs, including psychosocial aspects of living with HIV, and involved experts in the areas of infectious diseases, public health and psychiatry as well as community representatives.

All patients aged eighteen years and above were invited to answer the questionnaire annually on either a computerized or paper version at the outpatient clinic. The Questionnaire was used to improve the consultative call between members of the HIV-team and the patient. Those patients who were illiterate or who did not understand Swedish or English responded with the help of an interpreter.

**Fig 1:**
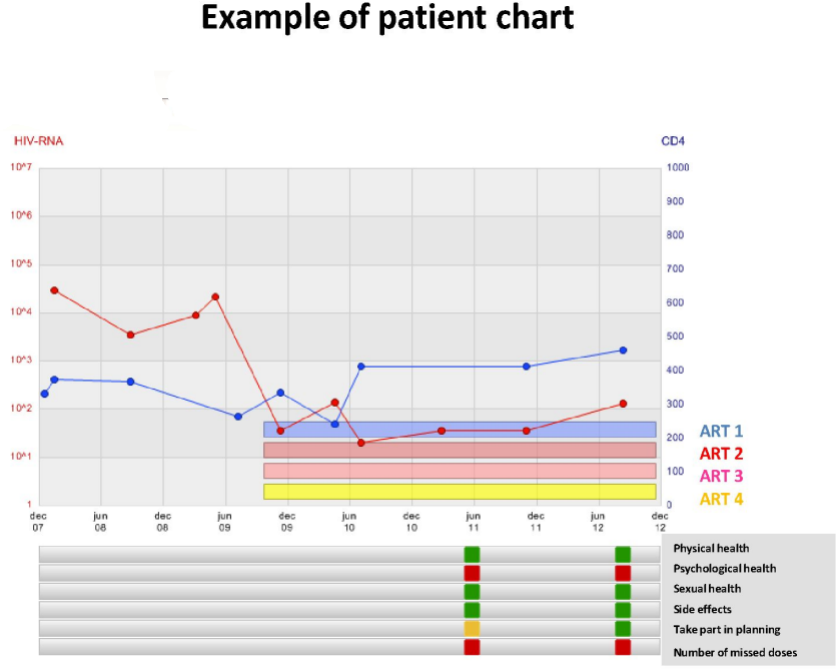
Example of a patient chart in the decision tool InfCareHIV. A green light is shown when patients report that they are satisfied or very satisfied in questions 1–3, they are not troubled by side effects, they report always feeling involved in their care and they do not miss doses. A yellow light is shown when patients are rather satisfied or rather unsatisfied in questions 1–3, they are troubled by side effects or they report sometimes feeling involved in their care. A red light is shown when patients are very unsatisfied or rather unsatisfied in questions 1–3, when they are very troubled or rather troubled by side effects, when they have missed one or more doses of ART in the past week or they report that they are seldom or never involved in the planning and realization of their HIV care and treatment. ART 1 corresponds to PI, ART 2 and 3 to NRTI and ART 4 to PI booster.

The patient’s responses to all items except for satisfaction with care are shown in the decision support tool (Fig 1). The individual patient’s responses regarding satisfaction with care are not presented on the graph and are only accessible at group level to the care-givers. Between 2012 and 2014, 2,846 patients from 30 HIV units, corresponding to 44% of all active patients in InfCareHIV, responded to the Health Questionnaire at least once. We included all questionnaires with at least one answer, even if the questionnaire was not complete. Patients who have answered the questionnaire more than once have been analyzed only considering the last questionnaire except for a sub-analysis of trends of adherence and its determinants that included all data from the subset of patients who had answered the questionnaire more than once. To measure test-retest reliability, a subset of patients with a medically stable health condition from 2014 (n = 57) was asked to respond to the questionnaire at baseline and one month later.

### Statistical methods

Descriptive analyses included frequencies for categorical variables and mean, and standard deviation for continuous variables. Anova and chi-squared tests were used to compare numerical and categorical variables among the years of survey. Logistic regression modelling was used to assess determinants of optimal adherence (defined as no ART doses missed in the past week) and determinants of improved adherence within patients. Odds Ratios (OR), 95% Confidence interval and p-values were used to present the logistic regression model results. P-values < 0.05 were considered significant. Test-retest reliability was analyzed by calculating proportion of agreement, intra-class correlations (items 1, 2 and 3), κ (items 4b and d) and weighted κ (items 5 and 6) [21]. The agreement between patients’ responses on item 4a (on ART or not) and their recorded treatment status in InfCareHIV was calculated for all participants in the 2014 cohort (n = 1,321). Data analysis was performed using the STATA software 13 (Stata Corp. College Station, USA) and IBM SPSS Statistics, version 22 (IBM Corp).

### Ethical considerations

The InfCareHIV registry has ethical approval for studies on de-identified patient data (Regional Ethical Review Board, University of Gothenburg Dnr 532–11).

## Results

### General description of the Health Questionnaire study Cohort

During the study period 2012–2014, a total of 2,846 patients from 30 HIV units in Sweden answered the Health Questionnaire at least once, while 171 patients declined participation. Two thirds (67%) of the participants were men (Table 1). The mean age was 47 years. The most common estimated route of transmission was heterosexual (49%), followed by male-to-male sex (40%) and intra-venous drug use (5%). The majority of the participants were born outside Sweden (52%). However patients born in Sweden and patients with male-to-male sex as route of transmission were overrepresented with regard to responding to the Health Questionnaire (Table 1).

**Table 1:** Socio-demographic, treatment and satisfaction characteristics of the sample.

Characteristics	Total	2012	2013	2014	InfCareHIV demographic characteristics 2012
**Health Questionnaire study (n %)**	2,846 (100.0)	391 (13.7)	1,134 (39.9)	1,321 (46.4)	6,381 (100)
***Gender***					
Female	950 (33.4)	117 (29.9)	361 (31.8)	472 (35.7)	2,386 (37.4)
Male	1,896 (66.6)	274 (70.1)	773 (68.2)	849 (64.3)	3,990 (62.6)
***Mean Age (standard deviation)***	46.6 (11.6)	46.2 (10.9)	46.8 (11.5)	46.6 (11.9)	37.6 (11.7)
***Route of transmission***					
Heterosexual	1,373 (48.7)	169 (43.7)	518 (46.1)	686 (52.4)	3,208 (51.5)
Male-to-male sex	1,125 (39.9)	175 (45.2)	481 (42.8)	469 (35.8)	1,974 (31.7)
People with intravenous drug use	153 (5.4)	30 (7.8)	54 (4.8)	69 (5.3)	416 (6.7)
Blood-products	34 (1.2)	2 (0.5)	16 (1.4)	16 (1.2)	117 (1.9)
Mother to child transmission	41 (1.4)	3 (0.8)	15 (1.3)	23 (1.8)	171 (2.8)
Unknown/Other	94 (3.3)	8 (2.1)	39 (3.5)	47 (3.6)	340 (5.5)
***Country of birth***					
Sweden	1,378 (48.4)	203 (51.9)	562 (49.6)	613 (46.4)	4,495 (40.2)
Abroad	1,468 (51.6)	188 (48.1)	572 (50.4)	708 (53.6)	3,714 (59.8)
***Country of transmission***					
Sweden	1,182 (41.5)	191 (48.9)	491 (43.3)	500 (37.9)	2,068 (32.4)
Abroad	1,664 (58.5)	200 (51.1)	643 (56.7)	821 (62.1)	4,308 (67.6)
***On treatment***					**P-value**
No	184 (6.5)	34 (8.9)	73 (6.5)	77 (5.9)	0.117
Yes	2,635 (93.5)	250 (91.1)	1,054 (93.5)	1,231 (94.1)	
***Viral Load for all patients***					
< 50 copies/ml	2,548 (86.4)	318 (81.3)	973 (85.8)	1,167 (88.3)	0.001
≥ 50 copies/ml	388 (13.6)	73 (18.7)	161 (14.2)	154 (11.7)	
***Viral Load for patients on treatment >6 months***					
< 50 copies/ml	1934 (92.7)	241 (89.6)	760 (92.7)	933 (93.6)	0.082
≥ 50 copies/ml	152 (7.3)	28 (10.4)	60 (7.3)	64 (6.4)	
***Experienced side effects*** *					
No	1,841 (72.8)	192 (57.5)	744 (73.5)	905 (76.5)	<0.001
Yes	688 (27.2)	142 (42.5)	268 (26.5)	278 (23.5)	
***Doses missed last week*** *					
0	2,257 (86.9)	281 (81.9)	921 (88.9)	1,055 (86.6)	0.020
1–2	294 (11.3)	55 (16.0)	99 (9.6)	140 (11.5)	
3 or more	46 (1.8)	7 (2.0)	16 (1.5)	23 (1.9)	
***Satisfied with physical health (Likert scale1-6)***					
1–4	1,053 (37.4)	168 (43.1)	418 (37.3)	467 (35.8)	0.035
5–6	1,761 (62.6)	222 (56.9)	703 (62.7)	836 (64.2)	
***Satisfied with psychological well-being (Likert scale1-6)***					
1–4	1,141 (40.7)	173 (44.4)	458 (41.2)	510 (39.2)	0.169
5–6	1,162 (52.3)	217 (55.6)	653 (58.8)	792 (60.8)	
***Satisfied with quality of care (Likert scale1-6)***					
<6	553 (19.7)	102 (26.4)	200 (17.9)	251 (19.3)	0.001
6	2,255 (80.3)	285 (73.6)	917 (82.1)	1,053 (80.7)	
***Feel involved in planning HIV care (Likert scale 1–4)***					
1–3	145 (6.0)	29 (8.3)	55 (5.7)	61 (5.5)	0.126
4	2,289 (94.0)	319 (91.7)	913 (94.3)	1,057 (94.5)	

Socio-demographic, treatment and satisfaction characteristics of the 2,846 patients participating in the Health Questionnaire Study Cohort, by year of survey.

*Questions asked only to patients on treatment

### Treatment, side effects and adherence

Almost all patients in the Health Questionnaire cohort were on ART (93.5%). The percentage of patients who achieved viral suppression with VL <50 copies/ml increased significantly from 81% to 88% during the study period (p = 0.001). When only the patients on ART (treated >6 months) were considered, the proportion of patients with treatment success increased from 90% to 94%, but the results were no longer significant (p = 0.082). The proportion of patients experiencing side effects decreased by almost half from 2012 to 2014 (p<0.001), at the same time as the proportion of patients reporting optimal adherence increased from 82% to 87% (p = 0.02).

**Fig 2:**
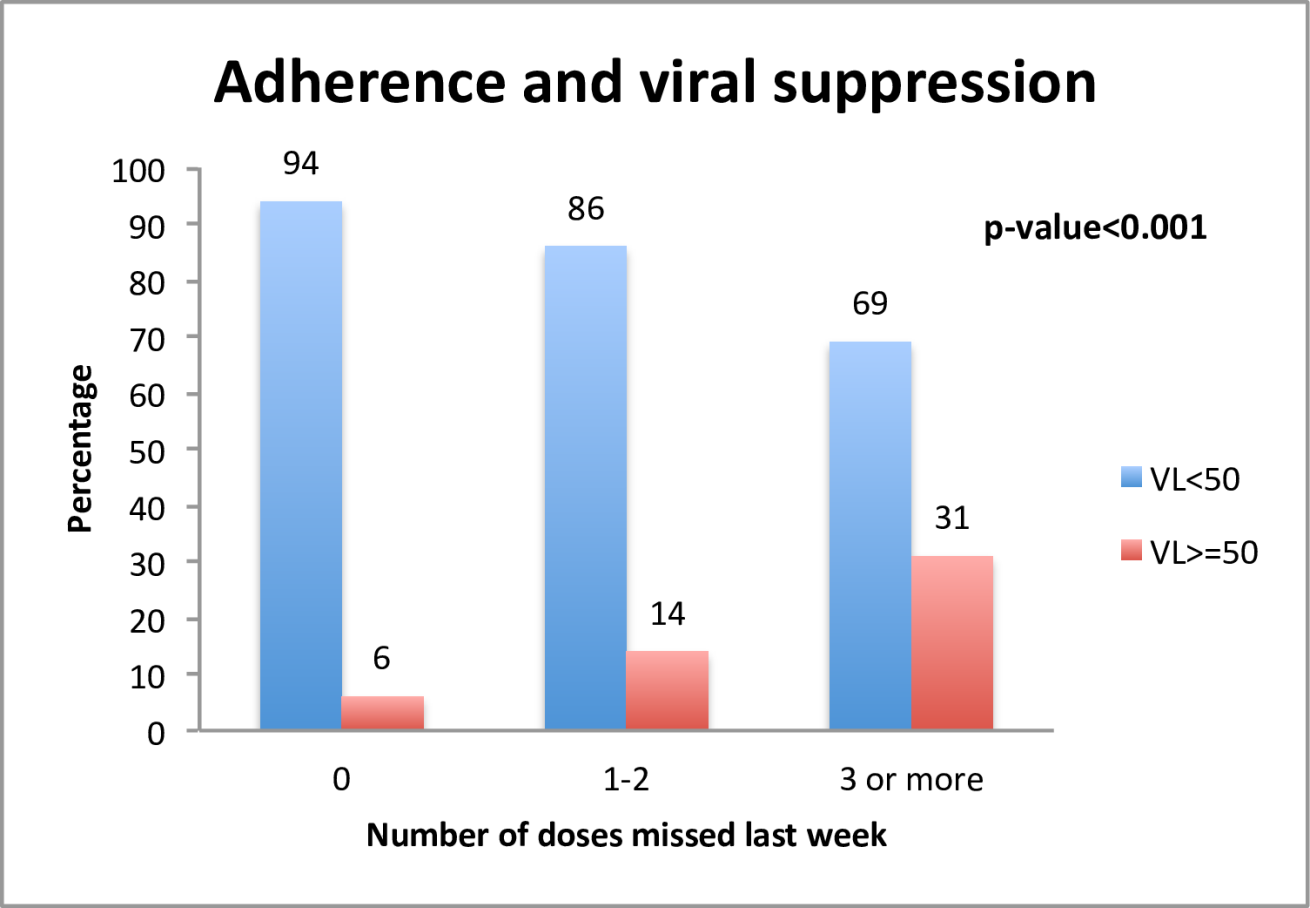
Percentage of patients virally suppressed stratified into different groups of adherence for the 2,086 patients participating in the Health Questionnaire Study Cohort, treated for more than six months.

Significant differences in viral load between different adherence groups were found regarding patients who were on ART > 6 months. Overall, 94% of the patients that reported not missing any doses had VL <50 copies/ml, while 86% and 69% of patients that reported missing 1–2 doses or ≥3 doses respectively had VL <50 copies/ml (p<0.001, Fig 2).

### Health and treatment satisfaction

Throughout the study period, 63% of patients were satisfied or very satisfied with their physical health, with the percentage increasing between 2012 and 2014 (p = 0.035). Fifty-two percent were satisfied or very satisfied with their psychological wellbeing. No significant differences in relation to gender or country of birth were seen in either of these two items. However, people with intravenous drug use (IDU) as route of transmission were significantly less satisfied with their physical and psychological wellbeing (p<0.001) compared to individuals with others routes of HIV transmission. Patients reporting being on ART were more satisfied with their psychological wellbeing compared to patients not taking ART (p<0.001), but there was no difference in reported physical health between these groups. Eighty percent of the patients were very satisfied with the quality of care they received, with numbers increasing over time (p = 0.001). Almost all the patients (94%) felt fully involved in the planning of their HIV care, with the percentage consistently over 90% throughout the whole study period.

### Determinants of adherence

**Fig 3:**
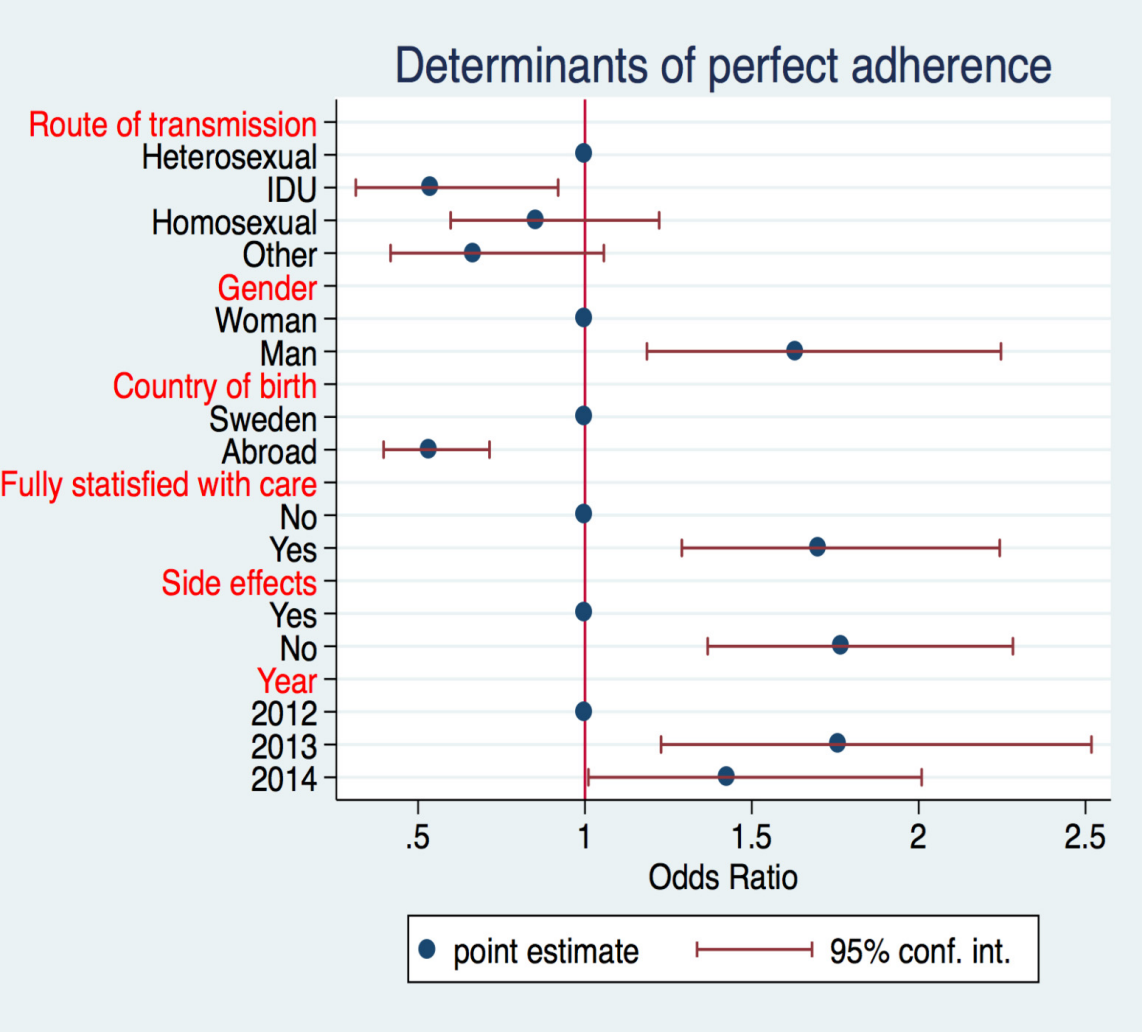
Logistic model assessing the determinants of adherence for the 2,086 patients participating in the Health Questionnaire study Cohort, treated for more than six months.

After dividing patients between adherent (reported no missed doses in the past week) and non-adherent (reported 1 or more missed doses in the past week), we assessed the correlation between variables in Table 1 as possible predictive factors of adherence. Results showed that the likelihood of being adherent was significantly lower for IDU compared to people with heterosexual transmission (OR: 0.52, 95% CI: 0.30–0.89) and for patients born abroad (OR: 0.59, 95% CI: 0.44–0.81) compared to people born in Sweden. It was however significantly higher for men (OR: 1.48, 95% CI: 1.07–2.05), patients fully satisfied with care (OR: 1.69, 95% CI: 1.28–2.22) and patients reporting no side effects (OR: 1.79, 95% CI: 1.38–2.31). Physical health and psychological wellbeing were not significant determinants of adherence. The likelihood of adherence also increased significantly compared to 2012, even after adjusting for all the variables in the model (Fig 3).

To study the characteristics of patients whose adherence improved during the study period, we made a longitudinal substudy of the patients who had answered the questionnaire more than once (n = 907) and who had reported missed doses in the first questionnaire (n = 104). A bivariate analysis with socio-demographic data from Table 1 was performed on the 104 patients which showed that patients born in Sweden were almost three times more likely (OR = 2.7, 95% CI: 1.1–6.9, p = 0.032) to improve their adherence during the study period than patients born in other countries.

### Reliability

Test-retest reliability was investigated by asking a subset of patients whose medical situation remained unchanged to respond to the questionnaire at baseline and then one month later; n = 57 of whom 50 responded to the full set of items relevant for them, depending on their treatment and experience of adverse events status. The three six-point Likert scale items regarding satisfaction with health showed agreement of 65%, 58% and 58% and ICC of 0.79, 0.84 and 0.87 for physical health (item 1), psychological health (item 2) and sexual health (item 3) items respectively. Item 4a (on ART or not) showed a test-retest agreement of 95% and when compared to the stated treatment status in InfCare the agreement was 99.5%. For item 4b (experiencing side effects or not) the test-retest agreement was 87% (κ 0.59; low variation of the responses, 74% reporting no side effects at both baseline and follow-up). Item 4c (severity of side effects) was only appropriate for seven of the participants (who had answered yes to item 4b on both occasions). Five of these seven had agreement in their test-retest responses and the remaining two had changed their response one step up or down on the four-point Likert scale (test-retest statistics not calculated due to few responses). For item 4d (number of missed doses in the past week) only two of the three response alternatives (no missed doses and 1–2 missed doses) were used with a 92% agreement (κ 0.67); although the one week recall period could make this item less stable in repeated measurements one month apart, there was a low variation of the responses, with 83% stating no missed doses at both baseline and follow-up. Item 5 (feeling involvement in care) showed an 84% agreement (weighted κ 0.48) and item 6 (satisfaction with care) showed an 83% agreement (weighted κ 0.37) although the variation of the responses on the scales was low with 74% and 78% respectively scoring highest possible at both baseline and follow-up (completely agree/completely agree and very satisfied/very satisfied, respectively).

## Discussion

In the Swedish national InfCareHIV cohort, which includes >99% of all known patients with HIV infection in Sweden, the number of patients on ART has increased every year. On December 31 2015, 6,605 of 6,946 patients (95.1%) were on ART. The number of patients that had been on treatment for at least six months was 6,395 and of those 6,053 (94.7%) had a viral load <50 copies/ml [22]. Sweden is the first country in Europe to reach the UNAIDS 90-90-90 targets for HIV care and treatment cascade [23]. However, the low levels of psychological well being in our cohort and the uneven distribution of satisfaction with care need further attention, with continued improvements in care to meet the individual needs of our patients.

After implementing the Health Questionnaire we found that self-reported adherence over a seven-day period demonstrated a high concordance with treatment outcome. Of patients who reported optimal adherence, 94% had a viral load <50 copies/ml, with the proportion significantly decreasing among patients missing 1–2 or 3 or more doses. The Health Questionnaire is a useful tool when analyzing cause of treatment failure. We found that the main determinants of optimal adherence, drawn from a multivariable model, were heterosexual transmission path, being born in Sweden, being male, not reporting side effects and being fully satisfied with care.

The discrepancy between reported non-optimal adherence and good treatment outcome can partly be explained by the time elapsed between answering the questionnaire and the date for VL measurement. That 69% of patients with VL <50 cop/ml reported missing >3 doses a week possibly reflects the fact that a protease inhibitor (PI) was used in the ART combination in more than 50% of the patients in the InfCareHIV Cohort, since PIs are safer in less adherent patients because of their reduced likelihood of developing resistance at virological failure [24]. This analysis also demonstrates that 6% of patients who reported optimal adherence had a viral load >50 copies/ml. Well known causes of full adherence and detectable virus are resistant virus, pharmacokinetic interference, transient viral blips and that self-report may overestimate adherence [25,26]. New results from the InfCareHIV cohort also show that subtype can influence viral affinity for PI used in ART. Reduced affinity for PI facilitated treatment failure and can be one more explanation of the gap between perfect adherence and treatment failure [27].

Longitudinal data from patients in the Health Questionnaire substudy cohort confirm that improved adherence among non-adherent patients is possible and that Swedish born patients were twice as likely to improve adherence compared to patients that had immigrated to Sweden after birth.

The significant relation between satisfaction with care and adherence has been confirmed in a previous cross-sectional national Swedish study [28]. Invariably, low adherence and lower satisfaction with care among immigrants compared to the Swedish born individuals indicate that the communication between the HIV-team and the patients needs improvement. The Health Questionnaire cohort includes patients from 220 nations and language issues are a major problem in daily care, even if translators are frequently used. Another reason, which may explain why immigrants are less satisfied with care, is previous experiences with the Swedish medical care. Sweden reports one of the highest proportions (74%) of migrants among late presenters compared to other European countries [29], many of them presenting an HIV related condition without an HIV test been done.

ART side effects are associated with non-adherence [30], which was confirmed in the present study where experiencing side effects was one of the main determinants for adherence. The proportion of patients experiencing side effects decreased over the years at the same time as the proportion of patients reporting optimal adherence increased. However, based on the InfCareHIV cohort, the frequency of patients being treated with third line PI and integrase inhibitors increased during the study period (data not shown). These drugs have fewer side effects and a simpler dose regime, and single dose regimens may enhance adherence [18].

We found the Health Questionnaire to be valid and reliable when used in ordinary clinical practice. The procedure used in its development included a review of the literature and the involvement of different stakeholders in the areas of infectious diseases, public health and psychiatry, as well as patient community representatives, to ensure the content validity of the measure. The test-retest reliability test performed on a subset of participants was evaluated according to Fayers and Machin [21] and showed a good to high test-retest agreement for satisfaction with physical, psychological and sexual health items. Although a high proportion of participants responded consistently regarding experience of side effects, adherence and involvement and satisfaction with care items, the test-retest statistics showed somewhat lower agreement, which could be explained by an asymmetric distribution of responses with a high proportion of the participants scoring towards one end of the scale and reporting being very satisfied [21]. This indicates that there is the potential to further develop the phrasing and response alternatives of these latter items to achieve more nuanced responses. When patients respond that they are on ART (item 4a), we see 99.5% conformity between prescribed medication in the InfCareHIV Database and the response. The responses to the question regarding adherence showed, in accordance with previous studies, an excellent concordance with the VL measures (p = 0.001) [11,12].

A limitation of the present study is that the ART regimens over time, including changes and dose interval, are not analyzed. Furthermore, it is not specified in the treatment whether a substance is given in a combination tablet or as a single generic. Studies concerning the relationships between adherence and generic antivirals versus brand combinations will be an important subject for further analyses.

We consider the large sample size and the large proportion o mfigrants participating in the study to be a strength, even if patients born in Sweden and homosexual men were slightly overrepresented. Since the demographic profile in 2014 was similar to the overall InfCareHIV cohort, our results seem to be generalizable.

## Conclusion

Since the results of this brief Health Questionnaire including only nine items are in concordance with other broader studies on adherence and QoL in HIV-infected individuals, we conclude that the instrument serves its purpose well, identifying patients at risk of treatment failure and patients in need of additional support and assessment.

## Supporting Information

S1 Fig Health Questionnaire used in the Swedish National Quality Assurance Registry InfCareHIV (PDF)
