# Supplementary material for: High Concordance between Self-Reported Adherence, Treatment Outcome and Satisfaction with Care Using a Nine-Item Health Questionnaire in InfCareHIV
Source: PLoS One. 2016 Jun 16;11(6):e0156916. doi: 10.1371/journal.pone.0156916 (PMC4911158; doi:10.1371/journal.pone.0156916)
Supplement: S1 Fig — (PDF) [file pone.0156916.s001.pdf]

S1 Fig. Health Questionnaire used in the Swedish National Quality Assurance Registry InfCareHIV

1. How satisfied are you with your physical health?

Very unsatisfied	Unsatisfied	Rather unsatisfied	Rather satisfied	Rather satisfied	Satisfied	Very satisfied
☐	☐	☐	☐	☐	☐	☐

2. How satisfied are you with your psychological wellbeing

Very unsatisfied	unsatisfied	Rather unsatisfied	Rather satisfied	Rather satisfied	Satisfied	Very satisfied
☐	☐	☐	☐	☐	☐	☐

3. How satisfied are you with your sexual life (regardless if you have sex with a partner or on your own)?

Very unsatisfied	Unsatisfied	Rather unsatisfied	Rather satisfied	Rather satisfied	Satisfied	Very satisfied
☐	☐	☐	☐	☐	☐	☐

If you are currently taking HIV medication, kindly answer the following questions:

4a. Do you experience any side effects?

Yes	No	If you answered yes, go to question 4b
☐	☐	

4b.To what extent are you troubled by medical side effects?

Very Troubled	Troubled	Rather troubled	Not very troubled	Not at all troubled
☐	☐	☐	☐	☐

4c. How many doses have you missed in the past seven days?

0	1-2	3 or more doses
☐	☐	☐

5. Do you feel involved in the planning and realization of your hiv care and treatment?

Never	Seldom	Sometimes	Always
☐	☐	☐	☐

6. How satisfied are you with the quality of care provided at your hiv clinic?

Very Unsatisfied	Unsatisfied Unsatisfied	Rather unsatisfied	Rather satisfied	Satisfied	Very satisfied
☐	☐	☐	☐	☐	☐
